# 4D flow evaluation of abnormal flow patterns with bicuspid aortic valve

**DOI:** 10.1186/1532-429X-11-S1-P184

**Published:** 2009-01-28

**Authors:** Michael D Hope, Alison K Meadows, Thomas A Hope, Karen G Ordovas, David Saloner, Gautham P Reddy, Marcus T Alley, Charles B Higgins

**Affiliations:** 1 UCSF, San Francisco, CA USA; 2 University of Washington, Seattle, WA USA; 3 Stanford, Palo Alto, CA USA

**Keywords:** Aortic Aneurysm, Thoracic Aorta, Bicuspid Aortic Valve, Helical Flow, Coronary Cusp

## Introduction

Bicuspid aortic valve (BAV) is the most common congenital heart defect and may account for more morbidity and mortality than all other congenital cardiac malformations combined. Two theories are commonly discussed for the elevated risk of aortic aneurysm and dissection in patients with BAV: 1) an increased hemodynamic load placed on the proximal aorta results in progressive dilatation and 2) a genetic or developmental abnormality in the proximal aortic tissue leads to weakness of the aortic wall.

## Purpose

This study utilizes 4D Flow to collect multidirectional blood flow velocity data in the thoracic aorta of patients with BAV. The goal of the study is to characterize altered flow patterns in these patients and uncover potential hemodynamic contributors to aneurysm and dissection formation.

## Methods

Time-resolved, 3D phase contrast MRI (4D Flow) was employed to assess thoracic aortic blood flow in 12 individuals: 8 patients with bicuspid aortic valve and 4 healthy subjects. The technique, which has been previously validated, was performed with parallel imaging after the standard clinical MR evaluation.

## Results

**Figure 1:**
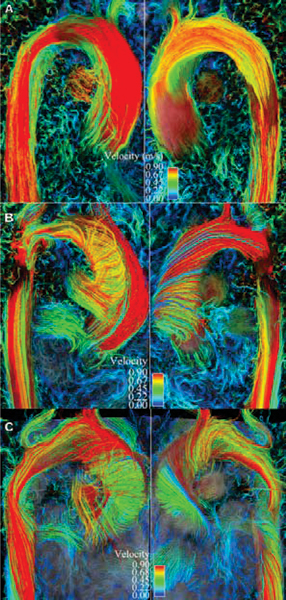
Figure 1

**Figure 2:**
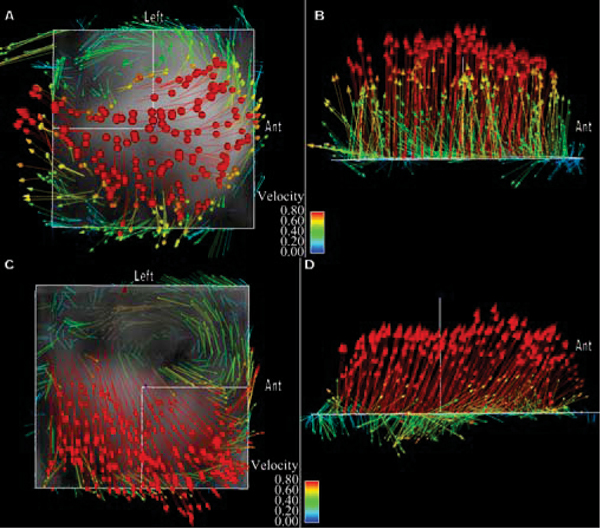
Figure 2

**Figure 3:**
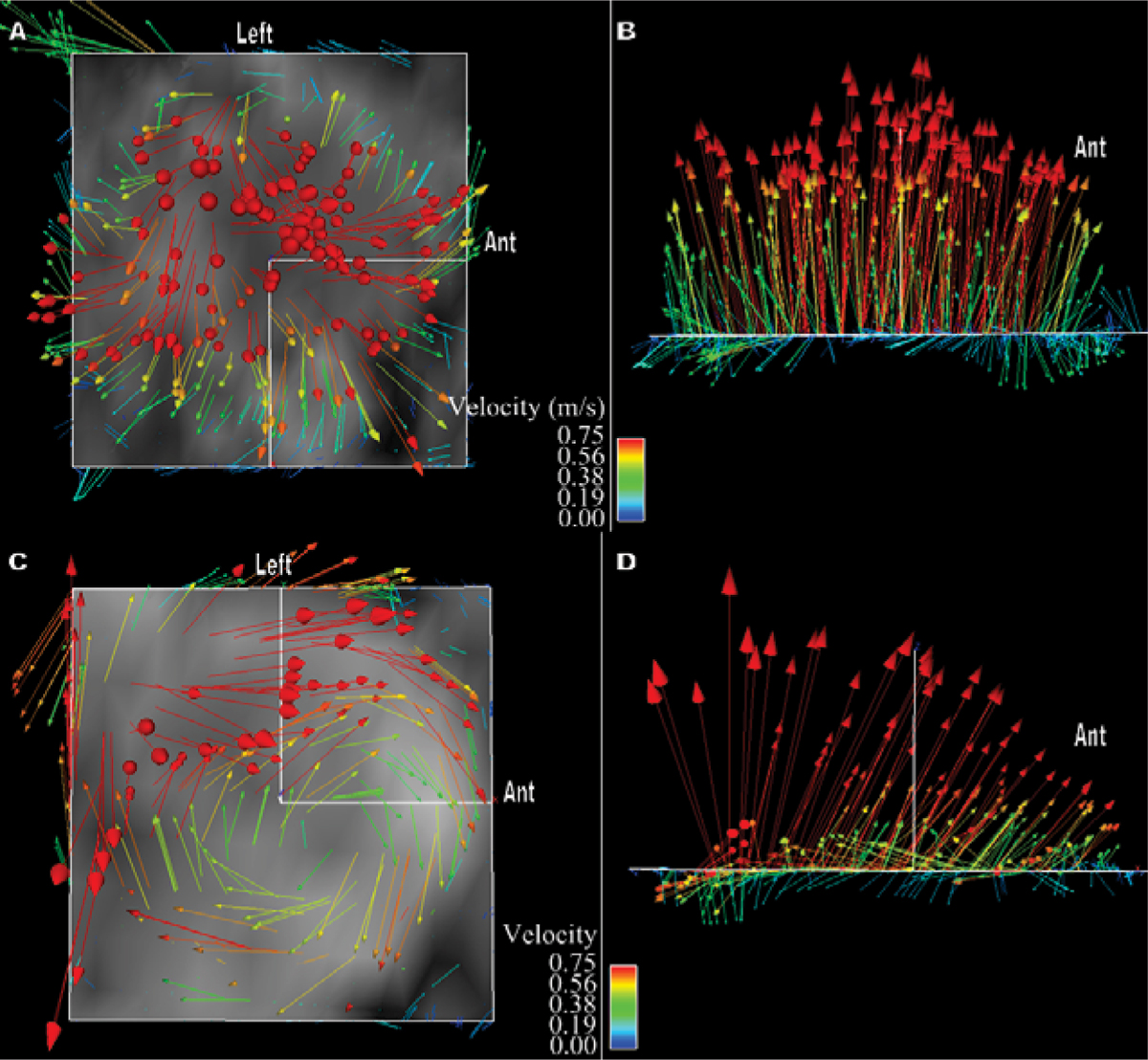
Figure 3

4D Flow evaluation of the ascending thoracic aorta revealed markedly abnormal systolic helical flow in 6 of 8 patients with bicuspid aortic valve. Five of these patients demonstrated an eccentric right anterior systolic jet and right-handed helical flow; at least two of these patients had fusion of the right and left coronary cusps, and three had aneurysms of the ascending aorta along with aortic stenosis and/or regurgitation. The other patient had a left posterior jet with left-handed helical flow, dilation of the aortic root and fusion of the right and noncoronary cusps. The two BAV patients with normal systolic flow in the thoracic aorta had central flow jets. Normal and abnormal systolic flow patterns are included as Figure 1: **1a** demonstrates normal flow with streamlines mapped onto a sagittal oblique plane viewed from right and left orientations respectively, **1b** shows an eccentric jet resulting in marked right-handed helical flow in a patient with BAV and aortic coarctation but without aneurysm, **1c** shows left-handed helical flow in a BAV patient with fusion of the right and noncoronary cusps. Evaluation of aortic flow jets in BAV patients and healthy subjects is included as Figures 2 and 3: **2a** and **b** represent systolic vectors from a plane orthogonal to the aorta just above the sinotubular junction in a healthy subject compared to **2c** and **d** which demonstrate an eccentric, right anterior flow jet in a patient with fusion of the right and left coronary cusps; **3a** and **b** show normal flow in a second healthy subject compared to **3c** and **d** which show a left posterior flow jet in a patient with fusion of the right and noncoronary cusps.

## Conclusion

Abnormal systolic helical flow is seen in the ascending thoracic aorta of patients with bicuspid aortic valve. Similar helical flow has been described in ascending aortic aneurysms associated with BAV, but we have demonstrated this flow pattern in two patients without aneurysm, suggesting that the pattern is not secondary to the dilated aorta, but may be implicated in the pathogenesis of aneurysm formation. The marked helical flow in the ascending aorta appears to be associated with eccentric flow jets in all 6 of our cases. In the single case of left-handed helical flow, fusion of the right and noncoronary cusps was found, a geometric configuration that may create a left posterior jet as we have demonstrated (Figure 3). Identification and characterization of eccentric flow jets in patients with BAV may help risk stratify for development of ascending aortic aneurysm and dissection.

